# Emerging role of N- and C-terminal interactions in stabilizing (β/α)_8_ fold with special emphasis on Family 10 xylanases

**DOI:** 10.5936/csbj.201209014

**Published:** 2012-11-01

**Authors:** Amit Bhardwaj, Pranjal Mahanta, Suryanarayanarao Ramakumar, Amit Ghosh, Sadhu Leelavathi, Vanga Siva Reddy

**Affiliations:** a Molecular Pathology Lab, International Centre for Genetic Engineering and Biotechnology, AREA Science Park, Padriciano 99, 34149, Trieste, Italy; b Department of Physics, Indian Institute of Science, Bangalore, India; c National Institute of Cholera and Enteric diseases, Kolkata, India; d Plant Transformation Group, International Centre for Genetic Engineering and Biotechnology, Aruna Asaf Ali Marg, New Delhi – 110067, India

**Keywords:** Family 10 Xylanase, TIM barrel fold, Thermal stability, Metal binding, Aromatic clusters, Protein engineering

## Abstract

Xylanases belong to an important class of industrial enzymes. Various xylanases have been purified and characterized from a plethora of organisms including bacteria, marine algae, plants, protozoans, insects, snails and crustaceans. Depending on the source, the enzymatic activity of xylanases varies considerably under various physico-chemical conditions such as temperature, pH, high salt and in the presence of proteases. Family 10 or glycosyl hydrolase 10 (GH10) xylanases are one of the well characterized and thoroughly studied classes of industrial enzymes. The TIM-barrel fold structure which is ubiquitous in nature is one of the characteristics of family 10 xylanases. Family 10 xylanases have been used as a “model system” due to their TIM-barrel fold to dissect and understand protein stability under various conditions. A better understanding of structure-stability-function relationships of family 10 xylanases allows one to apply these governing molecular rules to engineer other TIM-barrel fold proteins to improve their stability and retain function(s) under adverse conditions. In this review, we discuss the implications of N-and C-terminal interactions, observed in family 10 xylanases on protein stability under extreme conditions. The role of metal binding and aromatic clusters in protein stability is also discussed. Studying and understanding family 10 xylanase structure and function, can contribute to our protein engineering knowledge.

## Introduction

**Figure 1:**
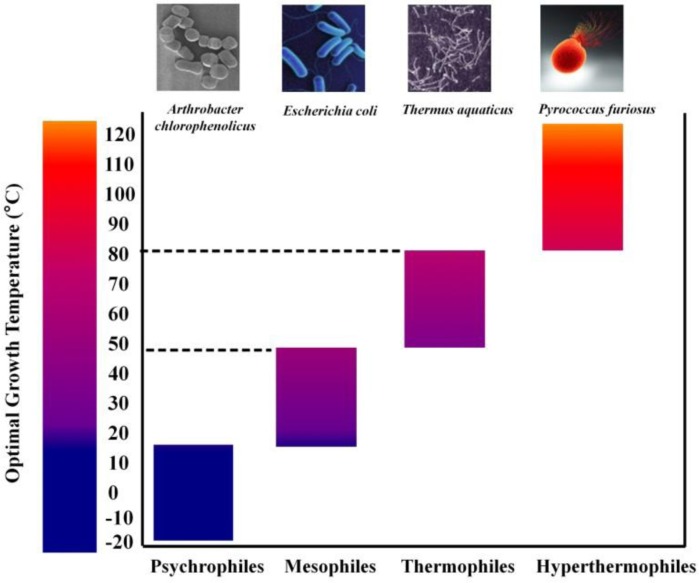
Optimal growth temperature range of various microorganisms.

Proteins play a vital role in the cellular metabolism of all living organisms. In general, enzymes are catalytically active globular proteins that control the rate of chemical reactions. This makes enzymes the most catalytically efficient bio-molecules, possessing high substrate selectivity and catalytic specificity for all the biological reactions. In nature, several organisms which include psychrophiles, thermophiles and hyperthermophiles have been found to thrive under extreme conditions such as low temperature or high temperature, pressure, high salinity, ionizing radiation etc. Organisms with optimal growth temperature (OGT) in the range of ≥ 80 °C are generally classified as hyperthermophiles, those with OGT in the range 45°C to 80°C are called thermophiles, those with OGT in the range 15°C to 45°C are mesophiles whereas psychrophiles have OGT ranging from -15°C to 10°C (Figure 1). Proteins are highly complex in nature and their structural integrity is maintained by a large number of interactions and a comparison between homologous proteins that have different stabilities may highlight specific interactions playing important role in protein stability. Sequence alignments, mutagenesis studies and crystal structure analyses have shown that differential stabilities of mesophlic and thermophilic xylanases are probably due to an array of minor modifications such as: an increased number of charged surface residues (1), an improved packing (2), an increase in the number of ionic interactions and hydrogen bonds (2, 3) and introduction of disulphide bridges particularly at the N- and C-termini or in the α-helix region (4, 5). Mutational studies involving T4 lysozyme and barnase have shown that protein stability is highly influenced by the nature and the position of the mutation where it was introduced (6, 7). Structural studies have shown that each class of enzymes has evolved specific adaptation strategies against extreme conditions and structural differences between the families are the basis for this difference in adaptation strategies (8, 2, 9). As a corollary, it is logical to say that each class of proteins have evolved its own mechanism to enhance protein stability under extreme conditions rather than converging on a single universal mechanism and hence necessary to identify the determinants of protein stability for each class of proteins. Interestingly, family 10 xylanases which possess TIM-barrel fold (present in approx. 10% of all the known enzymes) may be an excellent model system to address the structural and functional adaptation of an enzyme and recent studies have paved the way in this direction. In this regard, an extracellular endoxylanase, BSX, belonging to the GH10 family from an alkalophilic *Bacillus Sp*. NG-27 (GenBank ID: AAB70918.1; Uniprot ID: O30700; PDB ID: 2F8Q, 2FGL), is considered as a reference structure for structural analysis of GH10 stability (10). The crystal structure of BSX was solved at 2.2 Å (11) and has been extensively studied in our laboratory (12, 13). The present review highlights different stabilizing strategies adopted by the proteins of GH10 family to maintain their stability at high temperature. Besides, the critical role of N- and C-terminal interactions in the stability of GH10 family xylanases, a major subject of interest which has not been reviewed earlier has been covered in this article.

## Classification, Catalytic Mechanism, Sources and Applications of GH10 Xylanases

Xylanases or Endo-β-1,4-xylanases (EC 3.2.1.x) catalyze the endohydrolysis of 1, 4-β-D-xylosidic linkage in xylan, the predominant hemicellulose in the plant cell walls and comprises the second most abundant polysaccharide on earth. Xylanases are produced by many organisms including bacteria, marine algae, plants, protozoans, insects, snails and crustaceans (14). Most of the microbial xylanases are extracellular in nature as the large sized substrates cannot easily penetrate the cell wall. Extracellular xylanases expressed constitutively at low levels degrade the complex substrate (xylan) present outside the cell to generate xylo-oligomers which may be transported into the cell where they induce further xylanase synthesis (15, 16).

Plant cell walls are composed of three major polymeric constituents: cellulose, hemicellulose and lignin. The term “hemicellulose” was introduced by E. Schulze for the plant cell wall fractions which were isolated and extracted using dilute alkali solution (17). Hemicellulose (xylan) is the second most abundant renewable biomass and accounts for one third of all renewable organic carbon on earth (18). Xylan constitutes the major component of hemicelluloses, a complex of polymeric carbohydrate which includes xylan, mannan, galactan and arabinan. The principal monomers present in most of the hemicelluloses are D-xylose, D-mannose, D-galactose and D-arabinose. Most xylans occur as hetero-polysaccharides, containing different substitutions such as acetyl, arabinosyl and glucuronosyl residues at the backbone chain (19, 20). As a result of this heterogeneity and complexity, xylan requires a large variety of enzymes for its complete hydrolysis (19, 21, 22), leading to an abundance of diverse xylanases with different specificities, primary sequences and folds. Because of this variety, the proper classification of these enzymes on the basis of their substrate specificity alone became difficult. Initially, attempts were made to classify xylanases into two broad groups on the basis of their physiochemical properties (23) where the first group comprised xylanases with a low molecular weight (<30kDa) and basic pI and the second group had enzymes of high molecular weight (>30kDa) and acidic pI. A few years later, a more complete classification system based on primary structure comparisons of the catalytic domain was introduced (24).

**Figure 2:**
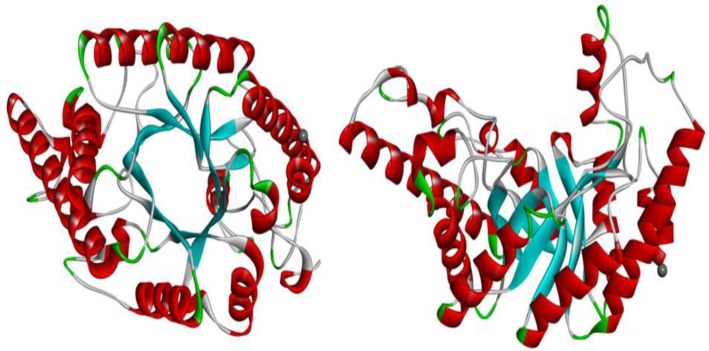
Overall structure of a family 10 xylanase (2F8Q) showing the typical TIM-barrel fold (a) top (b) side view.

This new system allows for the classification of glycosidases (EC 3.2.1.x) and groups of enzymes into families of related sequences (25). At present, 130 glycoside hydrolase families exist under the CAZy database (http://www.cazy.org/Glycoside-Hydrolases.html). Although, most xylanases have been classified as GH5, 8, 10 and 11; enzymes with xylanase activity are also found in different families: 7, 16, 26, 30, 43, 52, 62. However, some bi-functional and multi-domain enzymes with a demonstrated xylanase activity occur in GH7, 16, 43, 62 (26, 27). In addition, family 26 appear as endo-1,3-β-xylanase instead of endo-1,4-β-xylanases. Recently, on the basis of the arrangement of secondary structural element around the conserved (β/α)_8_-fold of the catalytic module; several GH5 enzymes have been reassigned into family 30 (28). So endo-1,4-β-xylanase activity containing distinct catalytic domain are restricted to families 5, 7, 8, 10, 11 and 43. Member of these families differ in their physico-chemical properties, structure and substrate specificities. Like GH10 xylanases, GH5 and GH30 xylanases display a (β/α)_8_-fold but GH5 xylanases are more specific to arabinoxylan (29) and GH30 xylanases are appendage-dependent that need free 4-O-methyl-D-glucuronosyl (MeGlcA) residues as side chain to be active (30, 31) whereas GH10 xylanases are much more versatile and have a broad substrate specificity. GH10 xylanases are highly active on short xylo-oligosaccharides, capable of hydrolysing aryl β-glycosides of xylobiose and xylotriose but not active on cellulose (32, 33). GH11 xylanases display a β-jelly-roll structure and are active on aryl-β-xylo-oligosaccharides but not on aryl-β-cello-oligosaccharides (32, 34). Enzymes in family 8 xylanases display a (α/α)_6_-fold and distinguish themselves from GH5, GH10 and GH11 xylanases by their inverting mechanism (35, 36). In contrast to GH5 xylanases, GH10 xylanases are a more closely related family and have a higher percentage of identical and spatially equivalent residues (37).Based on primary and three dimensional (3D) structures, most of the xylanases are generally classified into two major families of glycosyl hydrolases: family 10 (F) and family 11 (G) (38) The family 10 glycosyl hydrolase consists of endo-1, 4-β-xylanases (EC 3.2.1.8), endo-1, 3-β-xylanases (EC 3.2.1.32) and cellobiohydrolases (EC 3.2.1.91) (39) with majority are endo-1,4-β-xylanases. The members of this family have a high molecular mass and a structure of (β/α)_8_ barrel fold, also known as TIM-barrel fold resembling a ‘bowl’ (Figure 2). Previous studies based on crystal structure and kinetic analyses of activity on xylo-oligosaccharide have revealed that family 10 xylanases have five xylopyranose binding sites (40). Two glutamate moieties have been reported to act as catalytic residues of the enzymatic reaction, which proceeds via a double displacement mechanism (41, 42). GH10 endoxylanases are generally reported to be less selective and hence are able to attack various polysaccharides with different side chain modifications (32). These enzymes are highly active on short xylo-oligoscchardes and thus indicating small substrate binding sites (32). Primarily two different catalytic mechanisms have been proposed for the glycosyl hydrolases to hydrolyse glycosidic bonds: the retaining and the inverting mechanisms, both of which have already been discussed in great detail (38, 42–44). In brief, active site of retention and inverting enzymes is formed by two glutamic acid residues, which are approximately 5.5 Å and 9.5 – 7.5 Å apart respectively (45, 46), suggesting that the distance between the two catalytic residues is less constrained in inverting enzymes than in retaining enzymes. The retaining mechanism follows a double displacement reaction whereas the inverting mechanism follows a single displacement reaction. Family 10 xylanases catalyze hydrolysis through retaining mechanism.

Thermostable xylanases have been isolated from a variety of sources including terrestrial and marine solfataric fields, thermal springs, hot pools and self-heating decaying organic debris (47, 48, 49, 50). The majority of these belong to families 10 and 11. Family 10 xylanases have been isolated from various hyperthermophilic and thermophilic organisms including *Thermotoga sp*. (51, 52), *Bacillus stearothermophilus*T-6 (53), *Bacillus sp*. NG-27(8), *Bacillus sp. N16-5* (54), *Bacillus halodurans* (55), *Bacillus firmus* (56), *Caldicellulosiruptor sp*. (57), *Clostridium thermocellum* (58), *Rhodothermus marinus* (59) and *Thermoascus aurantiacus* (8, 60). A family 10 xylanase isolated from *Thermotoga sp*. Strain FjSS3-B.1 is one of the most thermostable xylanases reported with an optimum temperature for activity at 105 °C and pH 5.5 and a half life of 90 minutes at 95 °C (61). According to CAZy database, more than one hundred GH10 xylanase structures from over 20 different organisms have been solved to date (September 2012) and deposited in the Protein Data Bank (PDB). Although, many 3D catalytic domain structures of GH10 xylanases have been solved, only a few of them belongs to thermophilic xylanases. Table 1 shows the list of all GH10 thermophilic xylanases under the CAZy database.

**Table 1:** List of GH10 xylanases whose crystal structures are known to date.

Thermostable xylanases				

S. No	Name of the Protein	Organism	PDB ID	Uniprot ID
1	Alkaline endo-β-1,4-xylanase (Xyn10A)	*Bacillus halodurans S7*	2UWF	Q17TM8
2	Xylanase (BSX)	*Bacillus sp. NG-27*	2F8Q, 2FGL	O30700
3	Xylanase C / 10B (XylC; XynC; Xyl10B) (Xyn10B)	*Cellvibrio mixtus ATCC 12120*	1UQY, 1UQZ, UR1, 1UR2, 2CNC	O68541
4	Xylanase B (CELXYN; XynB)	*Clostridium stercorarium F-9*	2DEP	P40942
5	Xylanase Z / feruloyl esterase (XynZ; LX3)	*Clostridium thermocellum*	1XYZ	P10478.3
6	Xylanase Y / feruloyl esterase (XynY; LX5) (Xyn10B)	*Clostridium thermocellum YS*	2W5F, 2WYS, 2WZE	P51584
7	Xylanase (XlnC;X34)	*Emericella nidulans*	1TA3	Q00177
8	Xylanase (XynA2; IXT6) (intracellular)	*Geobacillus stearothermophilus T-6 NCIMB 40222*	1N82, 2Q8X, 3MS8, 3MSD, 3MSG, 3MUA, 3MUI	Q09LY9 Q9ZFM8
9	Xylanase T-6 (XynA;XT6)	*Geobacillus stearothermophilus T-6 NCIMB 40222*	1HIZ, 1R85, 1R86, 1RH7, 3MMD	P40943
10	Xylanase B (XynB;X-A) (Xyn10B)	*Paenibacillus barcinonensis BP-23*	3EMC, 3EMQ, 3EMZ	O69231
11	Xylanase A (XynA)	*Panicillum simplicissimum BT2246*	1B30, 1B31, 1B3V, 1B2W, 1B3X, 1B3Y, 1B3Z, 1BG4	P56588
12	Xylanase (Xys1;XysA)	*Streptomyces halstedii JM8*	1NQ6	Q59922
13	Xylanase A (XlnA) / IAF18 (Xyn10A)	*Streptomyces lividans 1326*	1E0V, 1E0W, 1E0X, 1OD8, 1V0K, 1V0L, 1V0M, 1V0N, 1XAS	P26514.2
14	β-1,4-xylanase (XynA;FXYN;SoXyn10A) (Xyn10A)	*Streptomyces olivaceoviridis E-86*	1ISV, 1ISW, 1ISX, 1ISY,1ISZ, 1IT0, 1V6U, 1V6V, 1V6W, 1V6X, 1V6Y, 2DIZ, 2D20, 2D22, 2D23, 2D24, 2G3I, 2G3J, 2G4F, 1XYF	Q7S198
15	Xylanase B (XynB;XylB;XTMB;TM0070) (Xyl10B)	*Thermotoga maritime MSB8*	1VBR, 1VBU	Q7WUM6 Q7WVV0 Q9WXS5
16	Xylanase A (XynA;XYLI;Xyn;TaXyn) (Xyn10A)	*Thermoascus aurantiacus IMI 216529*	1FXM, 1GOK, 1GOM, 1GOO, 1GOQ, 1GOR, 1I1W, 1I1X, IK6A, 1TAX, ITUX, 2BNJ, 3NYD, 3O2L	P23360
17	Endo- β-1,4-xylanase 10B (Tpet_0854) (Xyl10B)	*Thermotoga petrophila RKU-1*	3NIY, 3NJ3	A5IL00

**Mesostable xylanases**				

18	Xylanase B / 10A (Cex;Exg;XynB) (Xyn10A)	*Cellulomonas fimi ATCC 484*	1EXP, 1FH7, 1FH8, 1FH9, 1FHD, 1J01, 2EXO, 2HIS, 2XYL, 3CUF, 3CUG, 3CUH, 3CUI, 3CUJ	P07986.1 Q59277
19	Xylanase F / 10C (Xyl10C;CJA_3066) (Xyn10C)	*Cellvibrio japonicus Ueda107*	1US2, 1US3	B3PDA8 Q59675
20	Xylanase A / 10A (XynA;XylA;Xyl10A;CJA_2471) (Xyn10A)	*Cellvibrio japonicus Ueda107*	1CLX,1E5N,W2P,1W2V, 1W32,1W3H,1XYS	B3PKK3 P14768.2
21	Xylanase A/ III (XynIII;FoXyn10a;FOXG_17421) (Xyn10A)	*Fusarium oxysporum f. sp. Lycopersici 4287*	3U7B	B3A0S5
22	Methylglucuronoxylan xylanase A / xylanase A1 (XynA1;XynA;Pjdr2_0221)	*Paenibacillus sp. JDR-2*	3RDK, 3RO8,4E4P	C6CRV0 Q53I45
23	Endo- β-1,4-xylanase (XylE)	*Penicillum canescens VKPM F178*	4F8X	C3VEV9

Thermostable xylanases are widely used enzymes to replace/reduce toxic chlorine-containing chemicals in the paper pulp bleaching industry while being environmentally friendly (62). The global market for industrial enzymes was valued at 3.1 billon dollar in 2010 with an estimated value of $3.9 billion in 2011 and a projected value of $6 billion in 2016. The largest number of these enzymes belongs to the food and beverage enzymes with nearly $1.2 billion market in 2010 which is expected to reach $1.3 billion in 2011 and $2.1 billion in 2016 (http://www.bccresearch.com). Xylanases cover all the sections of industrial enzyme market of food and animal feed as well as technical enzymes and also constitute major commercial portion in hemicellulases. Sales figures for these are expected to increase as these enzymes are attracting increasing attention due to their potential and wide ranging applications in industrial processes. For example, xylanases from thermophilic *Bacillus sp. NCIM 59* increased the brightness of pulp by 2.5% (63). Thermo-alkaliphilic or even thermo- acidophilic xylanases may also be of use in bioconversion processes where a variety of treatments, including hot water and steam explosion, alkaline, solvent or acidic pre-treatments could be used prior to or simultaneously with the enzyme treatment (64, 65). Alkaliphilic xylanases would also be required for detergent applications where high pHs are typically used (66), while a thermostable xylanase would be beneficial in animal feeds if mixed before the pelleting process (typically carried out at 70°C – 95°C). One of the recent industrial uses of xylanases is in bio-ethanol production. Several countries have started special programs targeted towards developing biofuel production from renewable resources and examining the possibilities of biogas, bioethanol, biodiesel and fuel cell (67). Xylanase, combined with several other hydrolases, such as ligninase, xylosidase and glucanase etc., has the potential for being used for the generation of biological fuels, such as ethanol and xylitol (a sugar alcohol used as a naturally occurring sugar substitute) from lignocellulosic biomass (62).

## Common structural features responsible for stability of TIM barrel (β/α)_8_ fold

The (β/α)_8_ TIM-barrel fold was first observed in triose phosphate isomerase (68). These (β/α)_8_-barrel enzymes are present in all enzyme classes except ligases and is the dominating class among all hydrolases. An interesting concept of “division of labour” has been proposed by dissecting the (β/α)_8_-barrel into a “catalytic face” (comprises C-terminal ends of β strands) and a “stability face” (comprises the loops between α helices and subsequent β strands) which makes it possible to modulate the catalytic activities by mutation without compromising stability (69). Various factors contributing to the folding and stability of the TIM barrel fold include packing of the β-strand residues in the barrel core (70), folding of TIM barrels by energy minimization (71), amino acid clustering pattern in TIM-barrel proteins (72), and the importance of long-range interactions to the stability of the TIM-barrel fold (73).

In a comprehensive study of 36 TIM barrel proteins, Gromiha et al., (74) examined the contribution of hydrophobic clusters and long range interactions in hydrophobic clusters to thermal stability (74). They found that most of the residues were arranged in hydrophobic clusters which might be providing stability to the proteins. In another study of 71 TIM barrel domains, almost 1000 stabilizing residues were identified and more than 430 stabilizing elements in the context of hydrophobicity, long range interactions and sequence conservation were observed (75). This study revealed that a few stabilizing residues were located within the N- and C-terminal loops and α- helices, whereas the majority of stabilizing residues were located in the β-sheets. Silverman and co-workers (76) suggested that amino acids in the β-sheets are crucial for stability, whereas the amino acids in α- helices and βα loops may not be very important for the stability of TIM barrel structures as they were highly mutable. The method of knowledge-based potential was used to analyze the stability of loops of the TIM barrel proteins using experimentally determined high resolution X-ray structures. Their finding suggested that αβ loops are more important than βα loops for the stability of the fold; although a few loops are affected more than the others (76, 77). These findings were supported by protein engineering experiments with TrpA and TrpF proteins (78).

Sequence and structural comparison of thermophilic and mesophilic xylanases indicated that although both are very similar (79), enhanced thermostability is probably the result of some minor modifications both at sequence and structural level. The structure of *T. aurantiacus* GH10 xylanase RTUX (1.11Å, 293K) and CTUX (0.89Å, 100K) was determined at two different temperatures and resolutions. Structural comparison of RTUX and CTUX from *T. aurantiacus* GH10 xylanase revealed the crystallographic evidence of the plasticity of salt bridges and the role of water mediated interactions at different temperatures (60). The salt bridge between R124 - E232 is, to a large extent, bidentate in RTUX whereas it is water-mediated in CTUX.

In another report, Xie et al., (80) described the structural basis of thermostability of an intracellular *Cellvibrio mixtus* xylanase, CmXyn10B, using forced protein evolution by error prone PCR (80).

The crystal structure of the CmXyn10B double mutant (A334V/G343D) showed that introduction of Val334 fills a cavity within the hydrophobic core of the xylanase, increasing the number of van der Waals interactions with the surrounding aromatic residues, while Oδ1 of Asp348 makes an additional hydrogen bond with the amide of Gly344, and Oδ2 of Asp348 interacts with the arabinofuranose side chain of the xylose moiety at the -2 subsite (80). Comparative analysis is also a useful tool to discriminate (hyper) thermophilic proteins from their mesophilic homologs. It also helps in understanding the underlying principle of protein structure-function relationships providing insights into the thermal stability of GH10 xylanases. A comparison of family 10 xylanase isolated from thermophilic (*Thermoascus aurantiacus* and *C. thermocellum*) and mesophilic sources highlighted the role of hydrophobic packing, interaction of helix dipole with charged side chains and increase in proline content at the N-terminal of helices in enzyme thermostability (8). The stability and catalytic activity of an enzyme are also affected by metal ions. Metal ions (such as Ca^2+^, Mg^2+^ etc.) play an important role in protein thermo-stabilization and catalytic processes. Almost 50% of TIM barrel proteins require metal ions for catalysis (81) and many proteins from the GH 10 family require metal ions for their stability and enzymatic activity. The XYLA from *Pseudomonas fluorescens* subsp is one of the first family 10 enzymes found to contain a calcium binding site (82). Its crystal structure suggested that Asp256, Asn261, Asp262, Asn253 and Asn258 formed a putative calcium binding domain. Three mutants of XYLA containing D256A, N261A and D262A single mutations were generated. Additionally, a fourth mutant of XYLA was also generated where all the three single mutations were combined (82). Biophysical characterization of all these enzymes showed that the removal of calcium from XYLA (native) resulted in 6°C drop in the T_m_. Calcium binding was also found to provide stability against chymotrypsin at concentrations of ≥ 1 mM Ca^+2^. This indicates that the initiation site for protease degradation is within the calcium binding domain in loop7 of XYLA and that the increased protease susceptibility of mutants indicated that the enhanced flexibility of loop7 was due to its inability to accommodate Ca^+2^. Abou-Hachem et al. (59) also have described the role of a tightly bound Ca^+2^ ion in stabilizing the modular family 10 xylanase from the thermophilic bacterium *Rhodothermus marinus* (59). In a study of Carbohydrate Binding Module CBM4-2 of xylanase 10A (xyn10A) from *Rhodothermus marinus*, it was seen that the binding of Ca^+2^ increased the unfolding temperature of the protein by 23 °C (83).The crystal structure analysis of BSX revealed a metal binding site (Mg^+2^) at the C-terminal end of the catalytic domain (11). The Mg^+2^ ion is coordinated by two side-chain oxygen atoms from Asn292 and Asp354, a main chain carbonyl oxygen atom of Arg351 with four water molecules. The biochemical analysis showed that catalytic activity of BSX increased in the presence of Mg^+2^ in a concentration dependent manner (11). The bound Mg^+2^ ion presumably provides additional structural stability to the C-terminal region of the enzyme, particularly the last secondary structural element (α8) as two of the metal-coordinating residues, Arg351 and Asp354 (the C-terminal residue), belong to α8. In the xylosaccharide bound BSX crystal structure, another metal binding site was observed with different specificities, which could be responsible for reduced enzymatic activity at high Mg^+2^ concentration.

## N- and C-terminal contacts and protein stability

In general, loops and N- and C- terminal regions have the highest mobility in a protein structure and are most likely the initiation sites of protein denaturation. The deletion of flexible loops and N and C-terminal regions can contribute to successful protein crystallization. It has been suggested that anchoring of loops to the rest of the protein and loop shortening increases the protein stability (84).

Chimeric xylanases in which the N- or C-terminus from a thermophilic TmxAcat xylanase and a hyperthermophilic TmxB family 10 xylanase (from *Thermotoga maritime* MSB8) were exchanged suggested the probable role of N and C-terminal interactions in the protein thermostability (85). It was shown that replacement of only one of the two terminal segments of TmxAcat with the corresponding segments from TmxB (i.e. chimeras BA5/A5B and AB5/B5A) resulted in destabilization. Most interestingly, significant stabilization of chimeric molecules was observed when both terminal segments belonged to the same parent enzyme (for example as in the case of BA4B and AB4A). This study showed that replacement of both terminals with those either from TmxAcat or TmxB resulted in a net gain in thermal stability.

In a mutant of family 10 xylanase (CjXyn10A) from the mesophilic bacterium *Cellvibrio japonicas* that contains D262N/A80T/R347C, it was shown that Cys347 (in the C-terminus of the mutant) makes a disulfide bond with gal-Cys10 (present at the N-terminus of native enzyme) (86). However, the N-terminal sequence containing gal-Cys-10 and the side chain of Cys347 were not visible in the crystal structure and hence could not be verified. To further investigate whether the N- to C-terminal disulfide bridge can be used as a general thermostabilization strategy, cysteine residues were introduced at the N- and C-terminal of xylanases from *C. thermocellum* (CtXyn10A) and *C. mixtus* (CmXyn10B) (86). It was shown that when the C-terminal cysteine mutation was combined with a cysteine inserted into an extended N-terminal sequence of CtXyn10A, the resultant enzyme was more thermostable than that of wild type CtXyn10A. Similarly, another study also showed that the introduction of an N to C-terminal disulfide bridge (L380/A26C) into a double mutant of *C. mixtus* (CmXyn10B) conferred a further ∼2 °C increase in the T_m_ value (80).

In an attempt to understand the occurrence of N- and C-terminal contact in proteins, Krishna et al., (87) did an extensive *in silico* analysis on protein structures available in the Protein Data Bank (PDB). This study showed that half of the single domain proteins in the database have the tendency to bring N- and C-terminal elements in direct contact. Additionally, 37% of these proteins have the probability of having at least two residues in each terminal element in contact with each other. Such terminal element interactions have been suggested to play some special role in initial protein folding, native state stability and final turnover (87).

Liu et al., (88) studied the effect of N- and C-terminal residues on the stability and activity of a family 10 xylanase (Xyn, 302aa) from *Aspergillus niger* (88). Based on sequence and structural alignment, Xyn was found to contain five disordered residues (DR) (Gln1, Ser-5) at the N terminus and one DR (Leu 302) at the C-terminus. To demonstrate the negative correlation between non-regular structure and optimum temperature for xylanase activity, five N-terminal DRs (XynΔN), one C-terminal DR (XynΔC) and the six bi-terminal DRs (XynΔNC) were deleted and all the three constructs were analyzed to determine the optimum temperature (T_opt_) for xylanase activity. The T_opt_ values for XynΔN, XynΔC and XynΔNC were found to be 6 °C lower, 6 °C higher and equivalent to that of Xyn, respectively. The half life (t_1/2_) value for XynΔN, XynΔC and XynΔNC were determined at 50 °C and found to be 2-, 3- and 4-fold longer than that of Xyn, respectively. This study showed that the deletion of N- and C-terminal (XynΔNC) residues had an opposing effect on Xyn T_opt,_ but had an additive effect on t_1/2_. The analysis of terminal DRs using a model structure of Xyn showed that the new N- and C-terminals can come closer to each other after the deletion of five N-terminal DRs and one C-terminal DR, suggesting that this close contact between the N- and C-terminal can provide additional compactness to the structure thereby making the deletion mutants more stable than native Xyn (88).More recently, the role of N- and C-terminal contact in the family 10 xylanase BSX was investigated through site-directed mutagenesis to assess protein folding and stability of these mutants under more than one extreme condition (12, 13). The *in silico* analysis of the crystal structure of native (PDB ID: 2F8Q) and xylosaccharide-bound BSX (PDB ID: 2FGL) revealed the presence of various partially exposed thermostabilizing residues at the N terminus of mature BSX (Val, Gln, Pro, Phe and Trp) (89, 90) which might be contributing in stability of BSX.The role of partially exposed Val1 in the stability of BSX was examined under poly extreme (which include high temperature, pH, presence of SDS and proteases) conditions by creating a series of mutants in which Val1 was either deleted or replaced with other amino acids with different side chains (12). Critical evaluation of these mutants in comparison to those of recombinant BSX revealed the role of N- and C-terminal hydrophobic interactions in compact packing and stabilization of the BSX. Of all the mutants, Val1Gly was the most sensitive mutant of BSX under all the tested poly extreme conditions. On the other hand, the Val1Leu mutant was found to be more stable than the native enzyme and was also more tolerant towards SDS denaturation and protease action. This study suggested that the N-terminus of BSX plays an important role in overall stability of the enzyme.

**Figure 3:**
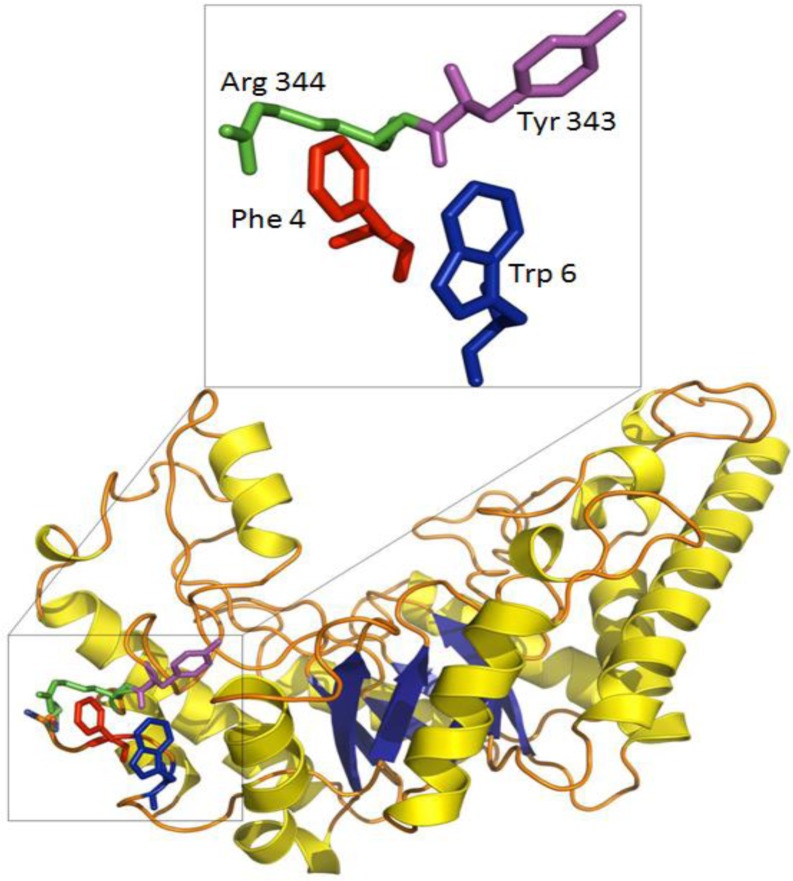
Various N- and C- terminal residues (in color) are shown in the side view of BSX crystal structure. A schematic diagram of residues involved in N- and C-terminal contacts is shown in enlarged window.

**Figure 4:**
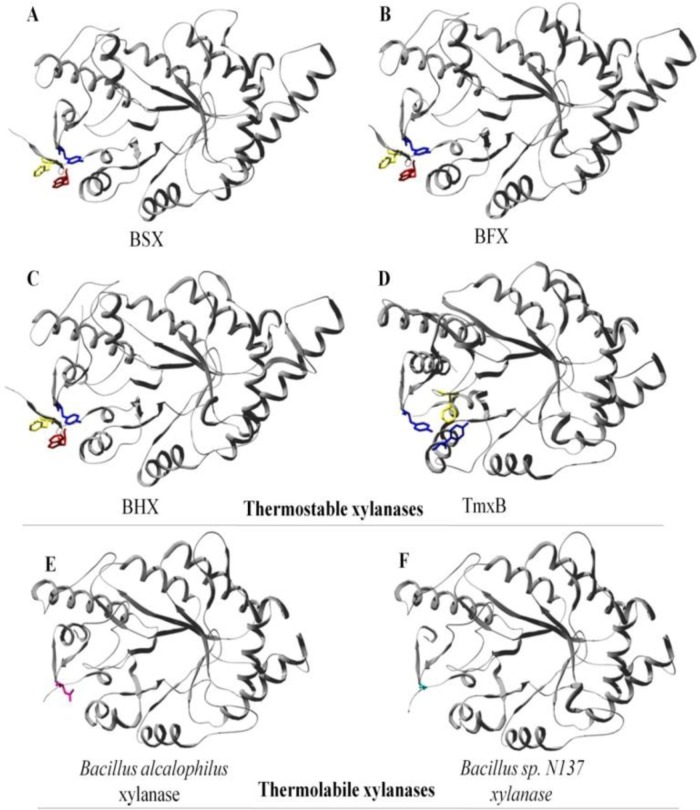
Interaction between N- and C-terminal regions. Structures of BFX (B), BHX (C) and TmxB (D) showed the presence of aromatic clusters equivalent to the studied F-W-Y cluster of BSX (A). E and F clearly show that xylanase from *Bacillus alcalophilus* and *Bacillus sp*. N137 does not contain any aromatic cluster to hold its N- and C-terminals together. This figure is adapted from Ref. (13).

The crystal structure of a Family 10 xylanase from *Bacillus sp*. NG-27 (BSX) showed that an aromatic cluster was involved in mediating the physical connection between the N- and C-terminal of BSX formed by two N-terminal residues (Phe4 and Trp6) and one C-terminal residue (Tyr343) (Figure 3) (13). Aromatic interactions are made up of a combination of forces which include electrostatic, hydrophobic and Van der Waals interactions (91). The contribution of a single aromatic interaction to the protein structure has been calculated to be between -0.6 to -1.3 kcal/mol (92) and has been reported to play important role in protein stability (93, 94, 95). In addition to aromatic cluster found in BSX, a couple of putative stabilizing cation-pi interactions involving Phe4-Arg344 and Trp6-Lys36 were also identified using *in silico* analysis (96). Cation-pi interaction between Phe4 –Arg344 is of special interest as this interaction is also involved in N- C-terminal interaction. Structural alignment of BSX revealed the presence of the same kind of N- to C-terminal aromatic cluster in a few more thermostable xylanases such as BHX (55), BFX (56) and TmxB (97), but these clusters were absent in thermolabile xylanases from *Bacillus alcalophilus* (Acc. No. AAQ99279) and *Bacillus* sp. N137 (98) (Figure 4). Interestingly, TmxB contains 46 aromatic residues and its crystal structure has shown that 38 out of 46 aromatic residues are arranged in five aromatic clusters. Cluster I is located around the N- and C- terminal regions connecting α1, α2, β1 and β8. However, no attempts have been made to experimentally test the role of aromatic residues involved in the formation of aromatic clusters in case of TmxB. In case of BSX, Alanine substitution mutants involving Phe4 (F4A), Trp6 (W6A) and Tyr343 (Y343A) were found to be sensitive to varying extents under all the conditions tested (13). This study neatly dissected the role of N- and C-terminal contacts in protein stability by creating and analyzing many mutants of BSX against various extreme conditions which would have been certainly a difficult task just on the basis of sequence/structure comparison alone.

The N- and C-terminal contacts have also been reported to play important role in protein stability for non- (β/α)_8_ fold containing proteins. One such important example comes from the cold shock proteins from the mesophile *Bacillus subtilis* (Bs-CspB, T_m_ = 53.9°C) and from the thermophile Bacillus Caldolyticus (Bc-Csp, T_m_ = 76.9°C) (99). Both Bs-CspB and Bc-Csp are small, monomeric proteins of 67 and 66 residues, respectively, does not contain any disulfide linkage and differ in sequence at 12 positions. The crystal structure of Bs-CspB (2.45 Å) (100) and Bc-Csp (1.17 Å) (101) revealed the almost identical backbone conformation and absence of any cofactors or tightly bound ligands. Further analysis showed that the thermostability of Bc-Csp has largely originated from the contribution of Arg3 (N-terminal) and Leu66 (C-terminal) residues and the equivalent positions are occupied by Glu residue in Bs-CspB. A double mutant of mesophilic Bs-CspB (Glu3Arg/ GLu66Leu) was created which removed the unfavourable electrostatic repulsion between Glu 3 (N-terminal) and Glu 66 (C-terminal) of wild type protein. Hence, the thermostability of Bc-Csp or thermolablie nature of Bs-CspB was found to be largely governed by the changes at only two positions, one near the N-terminus and the other near the C-terminus and hence revealed a simple and elegant way of protein evolution. Another such interesting example about the role of N- and C-terminus interaction in protein stability comes from an enzyme named as formylmethanofuran: tetrahydro-methanopterin formyltranferase (Ftr) isolated from a hyperthermophilic *Archaeon Methanopyrus* Kandleri (OGT 98 °C) (102). At high salt concentrations, Ftr assembles into a biologically active homotetrameric form and remains active and stable up to nearly 130°C. Crystal structure analysis of tetrameric Ftr revealed the role of multiple connections for higher structural rigidity involving N- and C-terminal segments of the structure. It was observed that N- and C-terminal residues, Met1 and Phe296 respectively were found to be tightly connected with each other and formed extended hydrophobic regions within the core and among subunits.

## Summary and Outlook

Extensive work in the field of protein engineering has shown that proteins attain stability by utilizing different stabilizing strategies which include: amino acid composition, stabilizing domains, hydrogen bonding, electrostatic interactions, hydrophobic interactions, cavity/core packing, oligomerization, metal binding, aromatic clusters and disulfide bonds (79, 81, 85, 93, 103, 104). A recent *in silico* analysis has shown that proteins have a general tendency to bring their N- and C-terminal in close proximity (87). In the case of the TIM-barrel fold, it has been shown that the protein sequence around the terminal region is less conserved compared to the sequence of the interior/core region. This makes it very difficult to predict these “terminal contacts” in the absence of any structural information. However, an increasing body of experimental evidence suggests a role for N- and C-terminus contact through aromatic stacking interactions in the stability of family 10 xylanases from *Bacillus sp*. NG-27 and *Aspergillus niger*. In addition to this, various family 10 xylanases have been observed to form N- and C-terminus contacts via aromatic cluster and covalent interactions using cysteine residues to enhance the overall protein stability. All these studies suggest that the proteins might have evolved the N- and C- terminus interactions as one of the strategies to stabilize their structures in a protein fold specific manner. It is tempting to speculate that besides family 10 xylanases, this might also be the case with other TIM-barrel fold containing proteins having N- and C- terminii in close proximity. It ought to be mentioned however, it is important to investigate many more proteins from diverse organisms to understand the biological significance of N- and C-terminal contacts that provide protein stability and help proteins to retain function under extreme conditions. Eventually such studies should enable one to design more stable proteins by taking into account various stabilizing interactions between N- and C-terminal ends.
